# Farnesol, a Sesquiterpene Alcohol in Herbal Plants, Exerts Anti-Inflammatory and Antiallergic Effects on Ovalbumin-Sensitized and -Challenged Asthmatic Mice

**DOI:** 10.1155/2015/387357

**Published:** 2015-04-19

**Authors:** Chi-Mei Ku, Jin-Yuarn Lin

**Affiliations:** Department of Food Science and Biotechnology, National Chung Hsing University, 250 Kuo Kuang Road, Taichung 40227, Taiwan

## Abstract

To investigate the effect of farnesol on allergic asthma, three farnesol doses were extra-added into AIN-76 feed consumed by ovalbumin- (OVA-) sensitized and -challenged mice continuously for 5 weeks, at approximately 5, 25, and 100 mg farnesol/kg, BW/day. The results showed that there were no significant differences in body weight, feed intake, and visceral organ weights between the farnesol supplementation and dietary control groups. Farnesol supplementation decreased interleukin (IL)-6/IL-10 level ratios in bronchoalveolar lavage fluid (BALF). Farnesol supplementation significantly (*P* < 0.05) restored the cytokine secretion ability of peritoneal macrophages that was suppressed as a result of OVA sensitization and challenge and slightly decreased tumor necrosis factor (TNF-*α*)/IL-10 cytokine secretion ratios. Farnesol supplementation slightly (*P* > 0.05) decreased IL-4 but significantly (*P* < 0.05) increased IL-2 levels secreted by the splenocytes in the presence of OVA, implying that farnesol might have a systemic antiallergic effect on allergic asthmatic mice. Farnesol supplementation significantly (*P* < 0.05) increased IL-10 levels secreted by the splenocytes in the presence of OVA, suggesting that farnesol might have an anti-inflammatory potential to allergic asthmatic mice. Overall, our results suggest that farnesol supplementation may be beneficial to improve the Th2-skewed allergic asthmatic inflammation.

## 1. Introduction

Asthma that is an allergic disease estimated to affect at least 300 million people worldwide has attracted much attention recently [1, 2]. Allergic asthma is a chronic airway inflammatory disease accompanied with increased inflammatory cell infiltration, lung inflammation, and airway hyperresponsiveness. Asthma results from airway inflammation involving a diversity of activated cells including mast cells, eosinophils, T-lymphocytes, neutrophils, macrophages, and epithelial cells. These cells are recruited to the site and release proinflammatory cytokine mediators that augment and regulate airway inflammation, resulting in airway hyperresponsiveness responsible for the chronic symptoms of dyspnea, wheezing, and chest tightness [3]. Airway inflammation results in denudation of bronchial epithelium and thickening of subepithelial basement membrane due to deposition of collagen. In addition, severe asthma has been characterized by occlusion of the bronchial lumen by mucus, hyperplasia and hypertrophy of the bronchial smooth muscle, and goblet cell hyperplasia [3]. Therefore, the levels of mucus (possibly mucin) and other proteins in the bronchial lumen may be selected as an inflammatory marker.

There are two distinct helper T lymphocytes, type 1 helper T (Th1) and Th2 cells that synthesize differential cytokines to influence immune responses. Interleukin (IL)-1, tumour necrosis factor (TNF), and IL-6 produced by Th1, Th2 lymphocytes, or other inflamed cells that highlight the way and trigger local inflammation within injured tissues can be roughly classified as proinflammatory cytokines [4]. In contrast, IL-10 produced by Th2 cells, T regulatory cells (Th3 cells), macrophages, and some B cells to inhibit Th1 synthesis and other cytokines and macrophage functions during the late inflammation phase are recognized as anti-inflammatory cytokines [5]. An imbalance in Th1/Th2 immune response patterns and pro/anti-inflammatory cytokines produced and generally accompanied with stress hormones may result in differential diseases, for example, persistent infections, severe immunosuppression, autoimmunity, allergy/atopy, tumour growth, and chronic graft-versus-host disease [6, 7]. Allergic asthma is characterized as a Th2-skewed disease. Regulation of the Th1/Th2 imbalance and anti/proinflammatory cytokine expression profiles in the host may avoid immune disorder diseases [8]. Potential phytochemicals from different food materials or herbs recently shed light on immunomodulation and may be beneficial for the corresponding human diseases [9].

Many drugs are used to treat asthma, such as inhaled corticosteroids, leukotriene inhibitors, mast cell stabilizers, and *β*2-adrenergic agonists that control the inflammation responses resulting from nitric oxide (NO), proinflammatory enzymes, and cytokines produced by macrophages [10]. The overaccumulation of proinflammatory mediators may cause severe damage to the heart, lung, and nerve system. At present, inhaled glucocorticoid is widely used to treat asthma; however, about 50% of asthma patients are not improved by the drug, which may induce adverse side effects, suggesting that alternative agents need to be developed [11]. Asthma exacerbations and early manifestations of the disease must be prevented to stop the diseases' evolution to severe asthma [12]. Therefore, natural traditional herbal medicines, health foods, and their possible active compounds show potential in treating asthma.

Among the possible active compounds against asthma, farnesol was found to have potential. Farnesol is a sesquiterpene alcohol that exists widely in fruits such as peaches, vegetables such as tomatoes and corn, herbs such as lemon grass and chamomile, and in the essential oils of ambrette seeds and citronella [13, 14]. Farnesol is found to alleviate massive inflammation, oxidative stress, and lung injury induced by the intratracheal instillation of cigarette smoke extract in rats [15]. Farnesol ameliorates 1,2-dimethylhydrazine induced oxidative stress, inflammation, and apoptotic in the colon of Wistar rats [14]. Farnesol has been shown to be potent in treating antimetabolic disorders, anti-inflammation, showing antioxidant, anticancer, and antibiotic effects [14, 16, 17]. Recently, we found that farnesol exhibited a relative Th1-inclination and anti-inflammatory property on immune cells that may be applied to improve Th2-skewed allergic asthmatic inflammation* in vivo* [18].

We hypothesized that farnesol has immunomodulation potential against allergic asthmatic inflammation. To validate this assumption, farnesol at different doses was administered to ovalbumin- (OVA-) sensitized and challenged mice for 5 weeks. The anti-inflammatory effects of farnesol supplementation on the experimental mice were determined.

## 2. Materials and Methods

### 2.1. Chemicals

**Figure 1:**
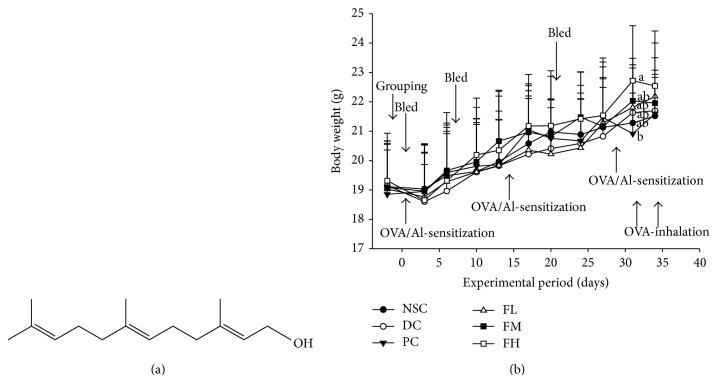
Farnesol structure (a) and its supplementation effects with different doses for 5 weeks on body weight changes of OVA/AL-sensitized and -challenged BALB/c asthmatic mice (b). Values are means ± SD (*n* = 12–15). Values among groups at the same experimental point not sharing a common small letter are significantly different (*P* < 0.05) from each other and assayed by one-way ANOVA, followed by Duncan's new multiple range test. NSC, nonsensitized control; DC, dietary control; PC, positive control (dexamethasone, 3 mg/kg BW, 0.3 mL/mouse by gavage); FL, low dose farnesol (0.003% in AIN-76 feed); FM, medium dose farnesol (0.017% in AIN-76 feed); FH, high dose farnesol (0.067% in AIN-76 feed).

Farnesol that is a sesquiterpene alcohol (C_15_H_26_O) in many plants was purchased at the highest available purity (>95%, a mixture of isomers) (Sigma, St. Louis, MO, USA). The chemical structure is shown in Figure 1(a).

### 2.2. Animal Grouping and Feeding

The experimental feed was prepared according to the American Institute of Nutrition AIN-76 recommendation that satisfies the nutritional requirement for mouse growth and varied only in farnesol composition [19]. Three farnesol doses, low dose (0.003%), medium dose (0.017%), and high dose (0.067%), were added to the AIN-76 feed (Table 1) [20]. Each feed was prepared by thoroughly mixing in farnesol and storing at −20°C. Approximately, 3 grams of AIN-76 feed was consumed per day by each individual mouse with 20 grams of body weight (BW). Farnesol low (FL), medium (FM), and high (FH) doses, respectively, corresponding to 5, 25, and 100 mg farnesol/kg BW/day, were designed for the experimental mice. It could be estimated that farnesol supplementation at the indicated doses might not produce significant energy* in vivo*. The energy contribution of each experimental diet was 67.5% from carbohydrate, 20.8% from protein, and 11.7% from fat. The calorie density of each diet was 3.85 Kcal/g. The animal use protocol listed below was reviewed and approved by the Institutional Animal Care and Use Committee (IACUC), National Chung Hsing University, Taiwan.

In our preliminary similar experiments, we found that the results from either male or female mice had same trend; however, female mice were more sensitive to treatments than male mice. Therefore, we selected female mice as our experimental mice in our following studies. The results from female mice in immunology have been accepted in many published papers. The female BALB/cByJNarl mice (7 weeks old) were obtained from the National Laboratory Animal Center, National Applied Research Laboratories, National Science Council in Taipei, and maintained in the Department of Food Science and Biotechnology at National Chung Hsing University College of Agriculture and Natural Resources in Taichung, Taiwan. The animal room was kept on a 12 h light and 12 h dark cycle. Constant temperature (25 ± 2°C) and humidity were maintained. The mice were housed and kept on a chow diet (laboratory standard diet, Diet MF 18, Oriental Yeast Co., Ltd., Osaka, Japan) to acclimatize for 1 week before feeding the experimental diet. After this equilibrium period, the mice were divided randomly into six groups (*n* = 15) varied by farnesol doses and sensitized treatments. The treatments were nonsensitized control (treated with phosphate-buffered saline (PBS) and alum (Al(OH)_3_), namely, PBS/AL, coded as NSC), dietary control (treated with OVA and alum, namely, OVA/AL, coded as DC), farnesol low dose (treated with OVA/AL, supplemented with low dose farnesol about 5 mg/kg BW/day, coded as FL), farnesol medium dose (treated with OVA/AL, supplemented with medium dose farnesol about 25 mg/kg BW/day, coded as FM), farnesol high dose (treated with OVA/AL, supplemented with high dose farnesol about 100 mg/kg BW/day, coded as FH), and positive control (treated with OVA/AL, treated with dexamethasone, coded as PC). NSC group is nonsensitized control group that is intraperitoneally injected with PBS and alum. NSC group is not suitable for a negative group in this study because it still induced mild inflammatory effects. Unfortunately, the experiment neglected to select normal mice that were not treated with any agents for a negative group. The initial average body weight of each group showed no significant differences among groups. Mice in each group were fed with the specified experimental diet* ad libitum* for 35 consecutive days. Mouse food intake and body weight were measured twice a week during the study period [20].

### 2.3. OVA-Sensitized and -Challenged Allergic Asthmatic Inflammation Mouse Model

The mice (8 weeks old) were sensitized and challenged to induce allergic airway inflammation. Mice were sensitized using an intraperitoneal injection (i.p.) of 0.2 mL alum-precipitated antigen containing 8 *μ*g of ovalbumin (OVA, albumin chicken egg grade III, Sigma-Aldrich Co., St. Louis, MO, USA) and 2 mg Al(OH)_3_ (Sigma-Aldrich Co., St. Louis, MO, USA) to induce primary immunity and started the specified experimental diets (at day 0). Two booster injections of this alum-OVA mixture were given 14 and 28 days later, respectively. Nonsensitized control mice received alum-phosphate-buffered saline (PBS, 137 mM NaCl (Wako Pure Chemical Industries, Ltd., Osaka, Japan), 2.7 mM KCl (Sigma-Aldrich Co., St. Louis, MO, USA), 8.1 mM Na_2_HPO_4_ (Sigma-Aldrich Co., Steinheim, Germany), 1.5 mM KH_2_PO_4_ (Sigma-Aldrich Co., St. Louis, MO, USA), pH 7.4, 0.2 *μ*m filtered) only. At days 31 and 34, the OVA-sensitized mice were challenged using aerosolized OVA at a concentration of 5 mg OVA per milliliter PBS for 60 min (namely, 9:00 am for 30 min and 4:00 pm for 30 min). The aerosolized OVA was produced using an ultrasonic nebulizer (sw918, Shinmed, Taipei, Taiwan). Nonsensitized control mice received only PBS [21]. Two hours before aerosolized OVA was administered, the PC group was treated with dexamethasone (DEX, 3 mg/kg BW, 0.3 mL/mouse, Sigma, St. Louis, MO, USA) by gavage to reduce allergic asthmatic inflammation [22]. Two days later (at day 36), all of the animals were anesthetized, exsanguinated using retroorbital venous plexus puncture, and immediately euthanized by CO_2_ inhalation (Figure 1(b)). The bronchoalveolar lavage fluid (BALF), peritoneal macrophages, spleen, and visceral organs were collected and assayed for cytokines and other inflammatory mediators [20].

### 2.4. BALF Collection

After the mice were euthanized, the airways and the lungs were immediately lavaged aseptically using a cannula through the trachea with 5 aliquots of 0.6 mL Hank's balanced salts solution (HBSS), free of ionized calcium and magnesium (HyClone Laboratories Inc., Logan, UT, USA). The BALF was centrifuged at 400 ×g for 10 min at 4°C. The supernatant (BALF) volume was determined and stored at −80°C for future assay [23].

### 2.5. Peritoneal Macrophage Isolation and Culture

After BALF collection, peritoneal macrophages from the experimental mice were collected according to the method described by Lin et al. [24]. Peritoneal cells were prepared by lavaging the peritoneal cavity with 2 aliquots of 5 mL sterile Hanks' balanced salts solution (HBSS) (50 mL of 10x HBSS (HyClone Laboratories Inc., Logan, UT), 2.5 mL of antibiotic-antimycotic solution (100x PSA) containing 10,000 units/mL of penicillin, 10 mg/mL of streptomycin, 25 *μ*g/mL of amphotericin B in 0.85% saline (Atlanta Biologicals Inc., Norcross, GA), 20 mL of 3% bovine serum albumin (BSA, Sigma-Aldrich Co., St. Louis, MO) in phosphate-buffered saline (PBS, 137 mM NaCl, 2.7 mM KCl, 8.1 mM Na_2_HPO_4_, 1.5 mM KH_2_PO_4_, pH 7.4, 0.20 *μ*m filtered), 2.5 mL of 7.5% NaHCO_3_ (Wako, Osaka, Japan), and 425 mL of sterile water) for a total of 10 mL through peritoneum. The peritoneal lavage fluid was centrifuged at 400 ×g for 10 min at 4°C. The cell pellets were collected and resuspended in tissue culture medium (TCM, a serum replacement; Celox Laboratories Inc., Lake Zurich, IL), suspended in a medium consisting of 10 mL TCM, 500 mL RPMI 1640 medium (Atlanta Biologicals Inc.), and 2.5 mL antibiotic-antimycotic solution (100x PSA) (Atlanta Biologicals Inc.). The peritoneal adherent cells (>90% of macrophages) from each animal were adjusted to 2 × 10^6^ cells/mL in TCM medium with a hemocytometer using the trypan blue dye exclusion method. The peritoneal macrophages (0.5 mL/well) in the absence or presence of lipopolysaccharide stimulus (LPS) (L-2654, Sigma-Aldrich Co., St. Louis, MO; at the final concentration of 2.5 *μ*g/mL, 0.5 mL/well) were cultured in 48-well plates. LPS, an endotoxin from Gram-negative bacteria, was selected to augment macrophages' inflammation* in vitro* [25, 26]. The plates were incubated at 37°C in a humidified incubator with 5% CO_2_ and 95% air for 48 h. The plates were then centrifuged at 400 ×g for 10 min to obtain the cell culture supernatants. The cell culture supernatants were collected for cytokine assay using enzyme-linked immunosorbent assay (ELISA).

### 2.6. Tissue Collection and Analysis

The thoracic and abdominal cavities of the experimental mice were opened aseptically. The organs such as heart, lung, liver, spleen, and kidney were removed and weighted. To evaluate the possible effects of farnesol supplementation on the body and visceral organs, the visceral organs weights in each experimental group were expressed as absolute and relative visceral organ weights, respectively [27]. The relative organ weight (%) was computed using the following equation: relative tissue (or organ) weight (%) = (individual tissue (or organ) weight (g)/body weight (g) of the experimental mouse) × 100.

### 2.7. Splenocytes Isolation and Cultures

The splenocytes were prepared by aseptically removing spleens from the experimental BALB/c mice. The spleen was ground with the flat bottom of a syringe piston to homogenize the splenocytes. Splenocytes were centrifuged at 400 ×g for 7 min at 25°C. The cell pellets were resuspended in 10 mL of red blood cell (RBC) lysis buffer (0.017 M Trizma Base (Sigma-Aldrich Co., St. Louis, MO, USA) and 0.144 M NH_4_Cl (Sigma-Aldrich Co., St. Louis, MO, USA) in deionized water, pH 7.2, 0.2 *μ*m filtered) for 3 minutes and centrifuged at 400 ×g for 7 min at 25°C. The cell pellets were washed with HBSS three times. Splenocytes were resuspended in TCM medium that contained 20% of TCM Serum Replacement (Protide Pharmaceuticals Inc., Illinois, USA) and 0.5% of Penicillin-Streptomycin Amphotericin B Solution in RPMI 1640 medium (HyClone, Utah, USA). The cells were counted with a hemocytometer using the trypan blue dye exclusion method. The cell density was adjusted to 1 × 10^7^ cells/mL in TCM medium. The isolated splenocyte suspensions (5 × 10^6^ cells/mL) were plated into 48-well plates and, respectively, incubated in the absence or presence of OVA (30 *μ*g/mL). The plates were incubated at 37°C in a humidified incubator with 5% CO_2_ and 95% air for 48 h. The cell culture supernatants were collected and stored at −80°C for cytokine assays.

### 2.8. Inflammatory Mediators and Markers Assay in BALF

#### 2.8.1. Nitric Oxide (NO) Assay

Eighty *μ*L aliquots of BALF samples and standards (0–100 *μ*M sodium nitrite (Sigma-Aldrich Co., St. Louis, MO, USA) dissolved in double distilled water) were pipetted into the 96-microplate wells (Nunc, Thermo Fisher Scientific, Rockford, IL, USA). One hundred sixty *μ*L aliquots of Griess reagent were then added into each well to develop the color. The Griess reagent was freshly prepared from Reagents A and B at a ratio of 1 : 1 (Reagent A: 1% (w/v) sulfanilamide (Sigma-Aldrich Co., St. Louis, MO, USA) dissolved in 5.0% (v/v) phosphoric acid (Riedel-de Haen, Seelze, Germany); Reagent B: 0.1% (w/v) N-(1-naphthyl)ethylenediamine dihydrochloride (Sigma-Aldrich Co., St. Louis, MO, USA) dissolved in deionized water). After incubation for 5 min, the plate was read on a plate reader (ELISA reader, ASYS Hitech, GmbH, Austria) at 550 nm. Using the standard curve, the NO concentration for each unknown sample was determined [20].

#### 2.8.2. Protein Level Assay

The BALF protein content was analyzed using bicinchoninic acid (BCA) protein assay (Thermo Scientific Inc., Rockford, USA), according to the accompanying instructions using a 96-well microtiter plate [23].

#### 2.8.3. Eotaxin Concentration

The BALF eotaxin concentration was determined using the mouse eotaxin sandwich ELISA kit (Quantikine M murine, R&D Systems, Minneapolis, MN, USA). The eotaxin concentration was assayed according to the manufacturer's instructions. The sensitivity of this assay was <3.9 pg/mL [20].

### 2.9. Measurement of Cytokine Levels in BALF and Peritoneal Macrophage Cultures Using ELISA

Cytokine (IL-4, IL-5, IL-2, IFN-*γ*, IL-1*β*, IL-6, TNF-*α*, and IL-10) levels in BALF, peritoneal macrophages, or splenocytes cultures were determined using sandwich ELISA kits, respectively. These cytokine concentrations were assayed according to the cytokine ELISA protocol of manufacturer's instructions (mouse DuoSet ELISA Development system, R&D Systems, Minneapolis, MN, USA). The sensitivity of these cytokine assays was <3.9 pg/mL [28].

### 2.10. Statistical Analysis

Data are expressed as mean ± SD. Data among groups were analyzed using analysis of variance (ANOVA), followed by Duncan's new multiple range test. Differences among groups were considered statistically significant if *P* < 0.05. Statistical tests were performed using SPSS version 12.0.

## 3. Results

### 3.1. Effects of Farnesol Supplementation on Intake and Growth of OVA-Sensitized and -Challenged Mice

The body weight and experimental procedure during the experimental period are given in Figure 1(b). The mean body weight of all experimental mice increased slightly as the experimental period was extended. There were no significant differences in mean body weight between the DC and other groups at each same experimental point. The feed efficiency and body weight changes in the experimental mice are shown in Table 2. There were no significant differences in the initial and final body weight, gain in body weight, feed intake, feed efficiency, and energy efficiency among groups. In the farnesol-treated mice, their mean feed intakes were 4.37 (FL), 4.81 (FM), and 4.70 g feed/day/mouse (FH), respectively. Three farnesol doses, low dose (0.003%), medium dose (0.017%), and high dose (0.067%), were added to the AIN-76 feed (Table 1). Therefore, the actual delivered doses to the farnesol-treated mice were 0.1311 (FL), 0.8177 (FM), and 3.149 mg farnesol/mouse/day (FH). Our results indicated that the farnesol treatment doses adopted in this study had no apparent toxic effects.

### 3.2. Effects of Farnesol Supplementation on Body and Visceral Organ Weights of OVA-Challenged Mice

The OVA-challenged mice were fed with different doses of farnesol for 5 weeks to evaluate farnesol effects on asthmatic inflammation. The results showed that farnesol supplementation did not significantly affect the liver, kidney, thymus, and heart weight among groups, indicating no toxic effect from farnesol treatments on the visceral organs (Table 3). However, OVA sensitization and challenge resulted in a slight increase (*P* > 0.05) in the spleen weight of BALB/c mice, indicating that a mild systemic inflammation was induced in the OVA-challenged mice. Importantly, DEX treatment (PC group) significantly decreased (*P* < 0.05) absolute and relative spleen and thymus weights compared to those in the DC group, indicating that DEX treatment effectively decreased systemic inflammation in the OVA-challenged mice. The spleen and thymus, particularly the spleen, are immune organs in the body that reflect systemic inflammation status. Farnesol treatments (FL, FM, and FH groups) just slightly, but not significantly (*P* > 0.05), decreased absolute and relative spleen and thymus weights, implying that farnesol treatments might alleviate systemic inflammation a little in the OVA-challenged mice. Interestingly, OVA sensitization and challenge caused a slight decrease (*P* > 0.05) in the epididymal fat weight of BALB/c mice, indicating a decrease in fat deposition resulting from allergic asthma in the OVA-challenged mice. However, farnesol and DEX treatment obviously increased the epididymal fat weight in the OVA-challenged mice, indicating that both farnesol and DEX treatments might alleviate the epididymal fat loss and improve nutrition status in the experimental mice.

### 3.3. Effects of Farnesol Supplementation on Cytokine and Inflammatory Mediator and Marker Levels in BALF of OVA-Challenged Mice

Table 4 shows proinflammatory and anti-inflammatory cytokine levels in BALF of OVA-sensitized and -challenged asthmatic mice. The results showed that there were no significant differences in IL-1*β*, IL-6, TNF-*α*, and IL-10 levels, as well as TNF-*α*/IL-10 (pro/anti-inflammatory) cytokine level ratios in BALF among the differential treatments, suggesting that there was just a mild inflammation induced in the airways and lungs in this animal model. Further comparison with proinflammatory and anti-inflammatory cytokine level ratios showed that both farnesol and DEX treatments slightly, but not significantly (*P* > 0.05), decreased IL-6/IL-10 (pro/anti-inflammatory ) cytokine level ratios in BALF. These results suggest that both farnesol and DEX treatments just slightly alleviate inflammation status in the lungs and airways of allergic asthmatic mice through decreasing pro/anti-inflammatory cytokine level ratios.

Table 5 shows the nitric oxide (NO), protein, and eotaxin levels in BALF. Unfortunately, there were no significant differences between the farnesol treatments and DC group. We presumed that there was just a mild inflammation induced in the airways and lungs in this animal model. Therefore, there is no significant difference in the eotaxin level in BALF between NSC and DC groups.

### 3.4. Effects of Farnesol Supplementation on Proinflammatory and Anti-Inflammatory Cytokine Secretion Levels in Peritoneal Macrophage Cultures from OVA-Challenged Mice

Table 6 shows the secretion levels of proinflammatory cytokines IL-1*β*, IL-6, and TNF-*α* as well as an anti-inflammatory cytokine IL-10 in peritoneal macrophage cultures from OVA-sensitized and -challenged mice through 5 weeks of feeding. In general, macrophages that are typical inflammatory cells should secret diverse cytokines when stimulated. However, our results showed that sensitization and challenge with OVA significantly inhibited (*P* < 0.05) proinflammatory and anti-inflammatory cytokine secretion levels by peritoneal macrophages in the absence or presence of LPS, implying that the decreased immunity might appear in allergic asthmatic mice. Importantly, both farnesol and DEX treatments significantly increased (*P* < 0.05) proinflammatory and anti-inflammatory cytokine secretion levels by peritoneal macrophages in the absence or presence of LPS as compared to those in the DC group, implying that both farnesol and DEX treatments increased immunity that might be suppressed as a result of allergic inflammation in allergic asthmatic mice. Further comparison with proinflammatory and anti-inflammatory cytokine secretion level ratios showed that farnesol treatments obviously decreased TNF-*α*/IL-10 (pro/anti-inflammatory) cytokine secretion level ratios. These results suggest that farnesol treatments alleviate systemic inflammation status through decreasing pro/anti-inflammatory cytokine secretion level ratios in peritoneal macrophages from allergic asthmatic mice. The farnesol treatment effects were better than DEX treatment (Table 6), implying that farnesol may be used to improve allergic inflammation in asthma patients in the future.

### 3.5. Effects of Farnesol Supplementation on Th1/Th2 Cytokine Secretion Levels in Splenocyte Cultures from OVA-Challenged Mice

**Figure 2:**
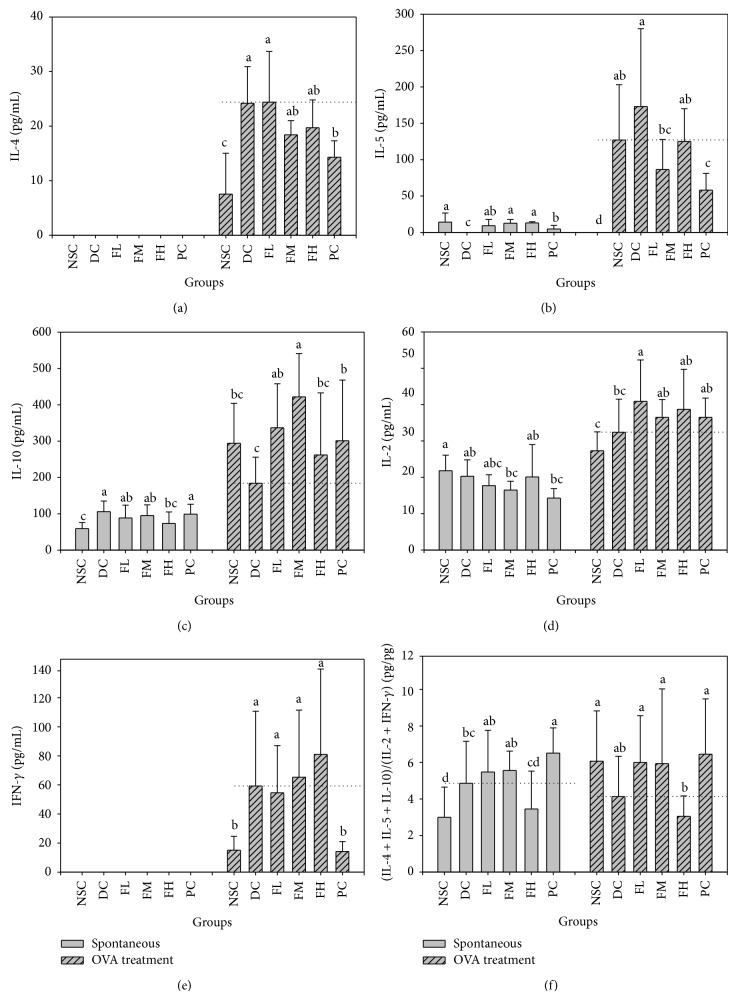
Farnesol supplementation effects with different doses for 5 weeks on IL-4 (a), IL-5 (b), IL-10 (c), IL-2 (d), IFN-*γ* (e), and (IL-4 + IL-5 + IL-10)/(IL-2 + IFN-*γ*) ratios (f) secreted by splenocytes of OVA/AL-sensitized and -challenged BALB/c asthmatic mice. Values are means ± SD (*n* = 12–15). Values among groups within the same treatment not sharing a common small letter are significantly different (*P* < 0.05) from each other and assayed by one-way ANOVA, followed by Duncan's new multiple range test. The limit of detection (LOD) of these kits used in this study was <3.9 pg/mL. NSC, nonsensitized control; DC, dietary control; PC, positive control (dexamethasone, 3 mg/kg BW, 0.3 mL/mouse by gavage); FL, low dose farnesol (0.003% in AIN-76 feed); FM, medium dose farnesol (0.017% in AIN-76 feed); FH, high dose farnesol (0.067% in AIN-76 feed).

To evaluate the effects of farnesol supplementation on systemic immune response in asthmatic subjects, Th1/Th2 cytokine levels in the splenocyte cultures from OVA-challenged mice were determined. The levels of Th1 (IL-2 and IFN-*γ*) and Th2 (IL-4, IL-5, and IL-10) cytokines in the splenocyte cultures in the absence or presence of OVA from OVA-challenged mice fed different experimental diets through 5 weeks are shown in Figure 2. The results showed that spontaneous cytokine secretion levels of Th1 (IL-2 and IFN-*γ*) and Th2 (IL-4 and IL-5), except IL-10, were too low to be detected. However, both Th2 (IL-4, IL-5, and IL-10) and Th1 (IL-2 and IFN-*γ*) cytokine secretion levels in splenocyte cultures in the presence of OVA were significantly (*P* < 0.05) increased as compared to those in the absence of OVA. All Th1 and Th2 except IL-10 cytokine secretion levels in DC group were significantly (*P* < 0.05) higher than those in NSC group. Moreover, spontaneous cytokine secretion profiles exhibited that Th2 (IL-4 + IL-5 + IL-10)/Th1 (IL-2 + IFN-*γ*) secretion ratios in DC group were significantly higher than those in NSC group (Figure 2(f)). These data indicated that OVA sensitization and challenge indeed induced a Th2-skewed systemic OVA-specific immune response, reflecting in the spleen. Farnesol supplementation slightly (*P* > 0.05) decreased IL-4 (a Th2 cytokine) (Figure 2(a)) but significantly (*P* < 0.05) increased IL-2 (a Th1 cytokine) (Figure 2(d)) levels secreted by the splenocytes in the presence of OVA, implying that farnesol supplementation might have an antiallergic effect on allergic asthmatic mice. The IL-10 level in NSC group was slightly higher than that in DC group, implying that the decreased immunity might appear in allergic asthmatic mice (Figure 2(c)). Furthermore, farnesol supplementation significantly (*P* < 0.05) increased IL-10 (a Th2 and anti-inflammatory cytokine) levels secreted by the splenocytes in the presence of OVA, suggesting that farnesol supplementation might also have an anti-inflammatory potential to allergic asthmatic mice (Figure 2(c)). Unfortunately, high dose farnesol supplementation (FH group) just slightly (*P* > 0.05) decreased Th2 (IL-4 + IL-5 + IL-10)/Th1 (IL-2 + IFN-*γ*) secretion ratios by the splenocytes in the absence or presence of OVA as compared to those in DC group (Figure 2(f)). Consequently, DEX treatments significantly (*P* < 0.05) decreased IL-4, IL-5 (Th2), and IFN-*γ* (Th1) but increased IL-10 (anti-inflammatory) cytokine secretion levels by the splenocytes in the presence of OVA as compared to those in DC group, implying that DEX treatments might inhibit immunity but have strong anti-inflammatory potential in allergic asthmatic mice (Figure 2). However, DEX treatments increased Th2 (IL-4 + IL-5 + IL-10)/Th1 (IL-2 + IFN-*γ*) secretion ratios by the splenocytes in the absence or presence of OVA as compared to those in DC group (Figure 2(f)), suggesting that DEX treatments might aggravate the Th2-skewed inclination in allergic asthmatic mice.

## 4. Discussion

In the present study, there was no significant influence of farnesol supplementations on intake and growth, indicating that the farnesol treatment doses adopted in this study had no apparent toxic effects (Table 2). Actual delivered doses to the farnesol-treated mice were 0.1311 (FL), 0.8177 (FM), and 3.149 mg farnesol/mouse/day (FH). Mean body weights of farnesol-treated mice calculated by their initial and final body weights during the experimental period were 20.7 (FL), 20.6 (FM), and 20.8 g/mouse (FH), respectively (Table 2). Thus, the actual delivered doses to the farnesol-treated mice were 0.1311 mg/20.7 g BW/day (FL), 0.8177 mg/20.6 g BW/day (FM), and 3.149 mg/20.8 g BW/day (FH), namely, 6, 40, and 151 mg/kg BW/day, respectively. The actual supplemented high dose of farnesol (151 mg/kg BW of mouse/day = 3.02 mg/20 g BW of mouse/day) to mice is equal to 1171 mg/day in humans according to an appropriate conversion ratio at 1 : 387.9 for mice (20 g) to human (70 kg) [20]. Horn et al. indicated that supplementing rats with 500 mg farnesol/kg BW by gavage for 28 days did not significantly influence their body weight, feed intake, and liver weights [29]. We found that supplementing experimental mice with farnesol at the indicated high doses for 5 weeks showed no apparent toxic side effects (Tables 2 and 3). Farnesol is a sesquiterpene alcohol that widely exists in fruits, vegetables, herbs, and essential oils [13, 14]. This study further provides farnesol safety data for food and possible pharmacological uses for anti-inflammatory and antiallergic effects. Our study suggests that farnesol supplementation at the indicated high dose is acceptable; however, the bioavailability of farnesol supplementation and its relative distribution in the body remain to be further clarified.

Asthma is a Th2-skewed allergic disease accompanied with systemic and airway inflammation [30]. In this study, we established a mild asthmatic animal model using OVA sensitization and challenge and evaluated farnesol anti-inflammatory and antiallergic effects* in vivo* [31]. Both Th2 (IL-4, IL-5, and IL-10) and Th1 (IL-2 and IFN-*γ*) cytokine secretion levels in splenocyte cultures in the presence of OVA were significantly (*P* < 0.05) increased as compared to those in the absence of OVA (Figure 2). In addition, all Th1 and Th2 except IL-10 cytokine secretion levels in DC group were significantly (*P* < 0.05) higher than those in NSC group. OVA sensitization and challenge markedly (*P* < 0.05) increased spontaneous secretion ratios of Th2 (IL-4 + IL-5 + IL-10)/Th1 (IL-2 + IFN-*γ*) by the splenocytes of the experimental mice (Figure 2(f)). The results evidenced that OVA sensitization and challenge successfully induced a Th2-skewed systemic OVA-specific immune response, reflecting in the spleen. We found that levels of NO, protein, and eotaxin in BALF showed no significant differences between the farnesol treatments and DC group (Table 5), indicating that the asthmatic animal model used in this study is still a mild model that did not cause severe injury or inflammation to the lungs and airways. Based on our results, OVA sensitization 3 times with subsequent 3 challenges is recommended to induce more severe asthmatic response in the lungs and airways. Eosinophils infiltration into the airways was detected but farnesol supplementation did not affect eosinophil numbers in BALF (data not shown). However, farnesol supplementation slightly (*P* > 0.05) decreased IL-4 (a Th2 cytokine) (Figure 2(a)) but significantly (*P* < 0.05) increased IL-2 (a Th1 cytokine) (Figure 2(d)) levels secreted by the splenocytes in the presence of OVA, implying that farnesol supplementation might have an antiallergic effect on Th2-skewed allergic asthmatic mice. High dose farnesol supplementation (FH group) slightly (*P* > 0.05), but not significantly, decreased Th2 (IL-4 + IL-5 + IL-10)/Th1 (IL-2 + IFN-*γ*) secretion ratios by the splenocytes in the absence or presence of OVA as compared to those in DC group, further suggesting a mild effect of farnesol supplementation against Th2 responses (Figure 2(f)). In addition, sera from the experimental mice were collected to measure antibody titers. The results showed that farnesol supplementation significantly increased (*P* < 0.05) OVA-specific IgG2a/IgE antibody titer ratios but decreased total IgE levels (data not shown). Our results evidenced that farnesol supplementation ameliorated allergic status and reversed Th2-skewed immune responses in the allergic asthmatic mice via decreasing serum OVA-specific IgE titers but increasing IgG2a/IgE titer ratios. Farnesol supplementation significantly (*P* < 0.05) reduced IL-4 levels in BALF that increased due to OVA sensitization and challenge, suggesting that farnesol supplementation may have potential to modulate Th1/Th2 immune balance toward Th1 pole in the airways and lungs (data not shown). Unfortunately, farnesol supplementation did not have significant effects on infiltrations of total cells and eosinophils into the airways and lungs (data not shown). Importantly, farnesol supplementation significantly (*P* < 0.05) increased IL-10 levels secreted by the splenocytes in the presence of OVA, implying that farnesol supplementation might also have an anti-inflammatory potential to allergic asthmatic mice (Figure 2(c)). Th2 cells are the major source of IL-10 production. However, Th2 cytokines, particularly IL-10, may inhibit the production of Th1 cytokines such as proinflammatory IL-1*β* and TNF-*α* cytokines. In addition, IL-10 is produced in late stage inflammation by immune effector cells to inhibit the synthesis of other cytokines. Therefore, IL-10 has been recognized as a Th2 and anti-inflammatory cytokine. Unfortunately, many cytokines production (IL-2, IL-4, IL-5, and IL-10) in farnesol-administered groups did not show dose-response phenomena (Figure 2). It is a universal mechanism to induce low- and high-zone tolerances of immunomodulation. Thus, the optimal dose for farnesol administration is difficult to ascertain. Farnesol administration should be carefully considered to achieve the best effect for various purposes. Our results suggested that the most effective dose of farnesol* in vivo* might be low dose administration for long term.

To compare the farnesol effects, dexamethasone (DEX), a potent synthetic member of the glucocorticoid family, was selected in this study as the positive control for its anti-inflammatory and immunosuppressant activities. We found that both farnesol supplementation and DEX treatment decreased enlarged spleen weights (Table 3), IL-6/IL-10 level ratios in BALF (Table 4), and TNF-*α*/IL-10 (pro/anti-inflammatory) cytokine secretion ratios by peritoneal macrophages (Table 6), indicating significant anti-inflammatory effects of farnesol and DEX on the airways and systemic inflammation. However, the anti-inflammatory effects of farnesol supplementation were much better than DEX treatment through decreasing TNF-*α*/IL-10 (pro/anti-inflammatory) cytokine secretion ratios by peritoneal macrophages (Table 6). To cure asthma, preventing the early manifestations of the disease and thus preventing its evolution into severe asthma are most important [12]. Our results suggest that farnesol may be used as a food supplement to prevent and improve allergic inflammation in asthma patients in the future. We found that OVA sensitization and challenge significantly inhibited IL-1*β*, IL-6, TNF-*α*, and IL-10 productions by peritoneal macrophages (Table 6); however, farnesol supplementation significantly restored the cytokine secretion levels, indicating that farnesol supplementation may enhance the inhibited immunity but inhibit inflammation in asthmatic mice. We assume that farnesol might exert its anti-inflammatory effect through modulating nuclear factor (NF)-*κ*B pathway [32]; however, its possible anti-inflammatory mechanisms remain to be further clarified. In addition, farnesol is considered to be a significant contact allergen and it was recommend that it should be included in a fragrance patch-test preparation and that its use should be regulated for consumer safety reasons [33]. The safety of dietary farnesol should be further studied.

## 5. Conclusions

Our results showed that actual farnesol supplementation at the indicated high dose of 151 mg/kg BW/day for 5 weeks had no toxic effect on the experimental mice. Farnesol supplementation decreased IL-6/IL-10 level ratios in BALF, suggesting an anti-inflammatory effect of farnesol on the lungs and airways. Farnesol supplementation significantly restored the secretion ability of peritoneal macrophages and slightly decreased TNF-*α*/IL-10 cytokine secretion ratios, indicating farnesol might enhance systemic immunity but inhibit inflammation in the lungs and airways in asthmatic mice. Farnesol supplementation slightly decreased IL-4 but significantly increased IL-2 levels secreted by the splenocytes in the presence of OVA, implying that farnesol supplementation might have a systemic antiallergic effect on allergic asthmatic mice. Furthermore, farnesol supplementation significantly increased IL-10 levels secreted by the splenocytes in the presence of OVA, suggesting that farnesol supplementation might also have an anti-inflammatory potential to allergic asthmatic mice.

## Figures and Tables

**Table 1:** AIN-76 and farnesol feed formula ingredients.

Ingredients (g/kg diet)	Formulas			
	AIN-76	FL	FM	FH
Casein	200	200	200	200
DL-methionine	3	3	3	3
Corn starch	150	150	150	150
Sucrose	500	500	500	500
Fiber	50	50	50	50
Soybean oil	50	50	50	50
Mineral mixture	35	35	35	35
Vitamin mixture	10	10	10	10
Choline bitartrate (41% choline)	2	2	2	2
Farnesol	0	38 *μ*L	188 *μ*L	753 *μ*L

Farnesol (%)	0	0.003	0.017	0.067
Suggested mg farnesol/kg BW/day	0	5	25	100

Calorie density (Kcal/g)	3.85			

Nutrients (% of total calories)				
Carbohydrate	67.5%			
Protein	20.8%			
Fat	11.7%			
Total	**100%**			

FL, low dose farnesol (0.003% in AIN-76 feed); FM, medium dose farnesol (0.017% in AIN-76 feed); FH, high dose farnesol (0.067% in AIN-76 feed).

**Table 2:** Effects of farnesol supplementation with different doses for 5 weeks on initial and final body weights and food and energy intake, as well as feed and energy efficiencies of OVA/AL-sensitized and -challenged BALB/c asthmatic mice.

Groups	*n*	Initial body weight	Final body weight	Body weight gain	Feed intake	Energy intake	Feed efficiency	Energy efficiency
		(g)	(g)	(g/d)	(g/d)	(kcal/day)	(g gain/100 g feed)	(g gain/100 kcal feed)
DC	13	19.1 ± 1.6	21.7 ± 0.8	0.07 ± 0.02	4.28 ± 0.62	16.5 ± 2.4	1.74 ± 0.57	0.45 ± 0.06
FL	14	19.1 ± 1.4	22.3 ± 1.5	0.09 ± 0.02	4.37 ± 0.46	16.8 ± 1.8	2.06 ± 0.49	0.54 ± 0.13
FM	12	19.2 ± 1.5	22.0 ± 1.4	0.08 ± 0.02	4.81 ± 0.27	18.5 ± 1.0	1.67 ± 0.27	0.43 ± 0.07
FH	14	19.2 ± 1.5	22.4 ± 1.8	0.09 ± 0.02	4.70 ± 0.34	18.1 ± 2.0	1.97 ± 0.38	0.51 ± 0.10
PC	14	19.2 ± 1.6	21.8 ± 0.9	0.07 ± 0.02	4.55 ± 0.31	17.5 ± 1.2	1.65 ± 0.60	0.43 ± 0.16

NSC	15	19.1 ± 1.4	21.5 ± 1.0	0.07 ± 0.02	3.82 ± 0.40	14.7 ± 1.5	1.81 ± 0.78	0.47 ± 0.20

Values are means ± SD. There are no significant differences among groups within same column assayed by one-way ANOVA, followed by Duncan's new multiple range test. NSC, nonsensitized control; DC, dietary control; PC, positive control; FL, low dose farnesol (0.003% in AIN-76 feed); FM, medium dose farnesol (0.017% in AIN-76 feed); FH, high dose farnesol (0.067% in AIN-76 feed).

**Table 3:** Effects of farnesol supplementation with different doses for 5 weeks on absolute and relative weights of visceral organs of OVA/AL-sensitized and -challenged BALB/c asthmatic mice.

Organ	Group	DC	FL	FM	FH	PC	NSC
	*n*	13	14	12	14	14	15
Spleen	ATW (g)	0.15 ± 0.03^a^	0.14 ± 0.01^a^	0.14 ± 0.02^a^	0.14 ± 0.03^a^	0.12 ± 0.01^b^	0.15 ± 0.03^a^
	RTW (%)	0.76 ± 0.16^a^	0.64 ± 0.21^ab^	0.68 ± 0.11^ab^	0.70 ± 0.15^ab^	0.58 ± 0.38^b^	0.72 ± 0.11^a^

Liver	ATW (g)	1.29 ± 0.11	1.33 ± 0.11	1.29 ± 0.13	1.29 ± 0.17	1.31 ± 0.11	1.36 ± 0.21
	RTW (%)	6.56 ± 0.32	6.51 ± 0.28	6.31 ± 0.51	6.25 ± 0.61	6.67 ± 0.08	6.61 ± 0.68

Kidney	ATW (g)	0.30 ± 0.03	0.30 ± 0.06	0.32 ± 0.02	0.31 ± 0.03	0.30 ± 0.02	0.30 ± 0.04
	RTW (%)	1.53 ± 0.12	1.44 ± 0.20	1.42 ± 0.46	1.52 ± 0.10	1.54 ± 0.05	1.49 ± 0.18

Heart	ATW (g)	0.12 ± 0.02	0.12 ± 0.01	0.12 ± 0.01	0.12 ± 0.01	0.12 ± 0.01	0.12 ± 0.01
	RTW (%)	0.61 ± 0.07	0.58 ± 0.06	0.61 ± 0.04	0.57 ± 0.18	0.61 ± 0.01	0.58 ± 0.03

Thymus	ATW (g)	0.02 ± 0.01^a^	0.02 ± 0.01^a^	0.02 ± 0.01^a^	0.02 ± 0.00^a^	0.01 ± 0.00^b^	0.03 ± 0.01^a^
	RTW (%)	0.11 ± 0.03^a^	0.12 ± 0.04^a^	0.11 ± 0.03^a^	0.12 ± 0.02^a^	0.04 ± 0.16^b^	0.13 ± 0.04^a^

Epididymal fat	ATW (g)	0.34 ± 0.13^b^	0.40 ± 0.08^ab^	0.40 ± 0.09^ab^	0.40 ± 0.12^ab^	0.47 ± 0.12^a^	0.42 ± 0.16^ab^
	RTW (%)	1.71 ± 0.60^b^	1.70 ± 0.79^b^	1.95 ± 0.41^ab^	1.94 ± 0.57^ab^	2.38 ± 0.06^a^	2.02 ± 0.78^ab^

Values are means ± SD (*n* = 12–15). Values within same row not sharing a common small letter are significantly different (*P* < 0.05) from each other and assayed by one-way ANOVA, followed by Duncan's new multiple range test. NSC, nonsensitized control; DC, dietary control; PC, positive control; FL, low dose farnesol (0.003% in AIN-76 feed); FM, medium dose farnesol (0.017% in AIN-76 feed); FH, high dose farnesol (0.067% in AIN-76 feed); ATW, absolute tissue weight; RTW, relative tissue weight.

**Table 4:** Effects of farnesol supplementation with different doses for 5 weeks on proinflammatory and anti-inflammatory cytokine levels in BALF of OVA/AL-sensitized and -challenged BALB/c asthmatic mice.

Groups	Proinflammatory and anti-inflammatory cytokines secreted in BALF				Pro/anti-inflammatory cytokine ratios	
	IL-1*β* (pg/mL)	IL-6 (pg/mL)	TNF-*α* (pg/mL)	IL-10 (pg/mL)	TNF-*α*/IL-10 (pg/pg)	IL-6/IL-10 (pg/pg)
DC	35.2 ± 22.8	34.6 ± 8.6	57.2 ± 13.0	60.0 ± 18.4	0.98 ± 0.27	0.60 ± 0.22^ab^
FL	32.8 ± 3.9	20.9 ± 18.3	50.6 ± 15.1	60.5 ± 14.8	0.84 ± 0.15	0.36 ± 0.24^b^
FM	34.3 ± 13.4	28.9 ± 22.0	48.9 ± 22.9	60.2 ± 24.2	0.81 ± 0.16	0.48 ± 0.32^ab^
FH	35.8 ± 5.6	23.4 ± 12.7	49.5 ± 9.6	55.5 ± 12.6	0.95 ± 0.10	0.37 ± 0.12^b^
PC	32.0 ± 3.3	21.4 ± 12.8	39.7 ± 7.9	51.8 ± 14.1	0.83 ± 0.19	0.40 ± 0.18^b^

NSC	28.3 ± 8.7	39.7 ± 21.0	54.3 ± 19.2	58.9 ± 15.7	0.91 ± 0.24	0.64 ± 0.26^a^

Values are means ± SD (*n* = 9–13). Values within same column not sharing a common small letter are significantly different (*P* < 0.05) from each other and assayed by one-way ANOVA, followed by Duncan's new multiple range test. The limit of detection (LOD) of these kits used in this study was <3.9 pg/mL. NSC, nonsensitized control; DC, dietary control; PC, positive control; FL, low dose farnesol (0.003% in AIN-76 feed); FM, medium dose farnesol (0.017% in AIN-76 feed); FH, high dose farnesol (0.067% in AIN-76 feed).

**Table 5:** Effects of farnesol supplementation with different doses for 5 weeks on inflammatory mediator and marker levels in BALF of OVA/AL-sensitized and -challenged BALB/c asthmatic mice.

Inflammatory mediator levels in BALF				
Groups	*n*	NO (*μ*M)	Protein (*μ*g/mL)	Eotaxin (pg/mL)
DC	9	5.89 ± 1.66	99.9 ± 12.4^abc^	278 ± 130^ab^
FL	11	5.22 ± 1.42	103 ± 22^ab^	382 ± 109^a^
FM	9	5.40 ± 1.33	106 ± 10^a^	323 ± 159^ab^
FH	11	6.42 ± 1.51	94.7 ± 12.7^abc^	298 ± 73^ab^
PC	11	5.68 ± 0.85	86.4 ± 7.6^c^	241 ± 79^b^

NSC	13	5.40 ± 1.42	90.0 ± 15.6^bc^	270 ± 96^ab^

Values are means ± SD. Values within same column not sharing a common small letter are significantly different (*P* < 0.05) from each other and assayed by one-way ANOVA, followed by Duncan's new multiple range test. NSC, nonsensitized control; DC, dietary control; PC, positive control; FL, low dose farnesol (0.003% in AIN-76 feed); FM, medium dose farnesol (0.017% in AIN-76 feed); FH, high dose farnesol (0.067% in AIN-76 feed).

**Table 6:** Effects of farnesol supplementation with different doses for 5 weeks on proinflammatory and anti-inflammatory cytokine secretions by peritoneal macrophages of OVA/AL-sensitized and -challenged BALB/c asthmatic mice.

Cytokines secreted by splenocytes	Groups	Treatment	
		Spon.	LPS
IL-1*β* (pg/mL)	DC	8.39 ± 3.88^b^	ND
	FL	14.0 ± 3.4^ab^	11.8 ± 8.4^ab^
	FM	11.9 ± 3.4^b^	18.5 ± 13.2^a^
	FH	12.4 ± 8.0^b^	18.5 ± 13.2^a^
	PC	11.5 ± 6.2^b^	8.3 ± 8.0^bc^
	**NSC**	**18.1 ± 10.6** ^a^	**18.4 ± 16.1** ^a^

IL-6 (pg/mL)	DC	159 ± 95^d^	537 ± 156^c^
	FL	270 ± 124^cd^	1047 ± 640^bc^
	FM	943 ± 528^a^	1915 ± 1066^a^
	FH	477 ± 278^bc^	1215 ± 786^b^
	PC	515 ± 292^bc^	1076 ± 619^bc^
	**NSC**	**595 ± 338** ^b^	**1231 ± 824** ^b^

TNF-*α* (pg/mL)	DC	192 ± 73^b^	332 ± 126^b^
	FL	250 ± 44^ab^	399 ± 74^ab^
	FM	271 ± 85^ab^	506 ± 96^a^
	FH	213 ± 141^ab^	356 ± 178^b^
	PC	301 ± 98^a^	505 ± 177^a^
	**NSC**	**301 ± 148** ^a^	**501 ± 215** ^a^

IL-10 (pg/mL)	DC	48.6 ± 35.1^c^	91.0 ± 41.0^b^
	FL	91.7 ± 36.7^ab^	166 ± 59^a^
	FM	129.3 ± 83.9^a^	193 ± 79^a^
	FH	65.9 ± 47.2^bc^	151 ± 80^a^
	PC	80.1 ± 46.0^bc^	159 ± 85^a^
	**NSC**	**80.9 ± 32.0** ^bc^	**150 ± 63** ^a^

TNF-*α*/IL-10 (pg/pg)	DC	3.89 ± 1.38^ab^	3.18 ± 1.36^ab^
	FL	2.46 ± 0.52^c^	2.54 ± 0.98^ab^
	FM	2.89 ± 1.60^bc^	2.13 ± 0.27^b^
	FH	3.18 ± 0.88^abc^	2.15 ± 1.05^b^
	PC	4.29 ± 1.98^a^	3.07 ± 1.04^ab^
	**NSC**	**3.63 ± 1.22** ^abc^	**3.52 ± 1.92** ^a^

Values are means ± SD (*n* = 12–15). Values within same column not sharing a common small letter are significantly different (*P* < 0.05) from each other and assayed by one-way ANOVA, followed by Duncan's new multiple range test. The LOD of these kits used in this study was <3.9 pg/mL. “ND” means not detectable. NSC, nonsensitized control; DC, dietary control; PC, positive control; FL, low dose farnesol (0.003% in AIN-76 feed); FM, medium dose farnesol (0.017% in AIN-76 feed); FH, high dose farnesol (0.067% in AIN-76 feed).
